# Pain and sensory detection threshold response to acupuncture is modulated by coping strategy and acupuncture sensation

**DOI:** 10.1186/1472-6882-14-324

**Published:** 2014-09-01

**Authors:** Jeungchan Lee, Vitaly Napadow, Kyungmo Park

**Affiliations:** Department of Biomedical Engineering, Kyung Hee University, Yongin Gyeonggi, 446-701 Republic of Korea; Martinos Center for Biomedical Imaging, Department of Radiology, Massachusetts General Hospital, Charlestown, MA 02129 USA; Department of Radiology, Logan University, Chesterfield, MO 63017 USA

**Keywords:** Coping strategy, Acupuncture, Acupuncture sensation, Pain, Sensory threshold

## Abstract

**Background:**

Acupuncture has been shown to reduce pain, and acupuncture-induced sensation may be important for this analgesia. In addition, cognitive coping strategies can influence sensory perception. However, the role of coping strategy on acupuncture modulation of pain and sensory thresholds, and the association between acupuncture sensation and these modulatory effects, is currently unknown.

**Methods:**

Electroacupuncture (EA) was applied at acupoints ST36 and GB39 of 61 healthy adults. Different coping conditions were experimentally designed to form an active coping strategy group (AC group), who thought they could control EA stimulation intensity, and a passive coping strategy group (PC group), who did not think they had such control. Importantly, neither group was actually able to control EA stimulus intensity. Quantitative sensory testing was performed before and after EA, and consisted of vibration (VDT), mechanical (MDT), warm (WDT), and cold (CDT) detection thresholds, and pressure (PPT), mechanical (MPT), heat (HPT) and cold (CPT) pain thresholds. Autonomic measures (e.g. skin conductance response, SCR) were also acquired to quantify physiological response to EA under different coping conditions. Subjects also reported the intensity of any acupuncture-induced sensations.

**Results:**

Coping strategy was induced with successful blinding in 58% of AC subjects. Compared to PC, AC showed greater SCR to EA. Under AC, EA reduced PPT and CPT. In the AC group, improved pain and sensory thresholds were correlated with acupuncture sensation (VDTchange vs. MI: r=0.58, CDTchange vs. tingling: r=0.53, CPTchange vs. tingling; r=0.55, CPTchange vs. dull; r=0.55). However, in the PC group, improved sensory thresholds were negatively correlated with acupuncture sensation (CDTchange vs. intensity sensitization: r=-0.52, WDTchange vs. fullness: r=-0.57).

**Conclusions:**

Our novel approach was able to successfully induce AC and PC strategies to EA stimulation. The interaction between psychological coping strategy and acupuncture sensation intensity can differentially modulate pain and sensory detection threshold response to EA. In a clinical context, our findings suggest that instructions given to the patient can significantly affect therapeutic outcomes and the relationship between acupuncture intensity and clinical response. Specifically, acupuncture analgesia can be enhanced by matching physical stimulation intensity with psychological coping strategy to acupuncture contexts.

**Trial registration:**

KCT0000905

**Electronic supplementary material:**

The online version of this article (doi:10.1186/1472-6882-14-324) contains supplementary material, which is available to authorized users.

## Background

Acupuncture treatment is known to reduce clinical pain, as evidenced in multiple clinical trials. For instance, a recent meta-analysis found that back and neck pain, osteoarthritis, headache, and shoulder pain were significantly improved after acupuncture treatment compared to sham acupuncture or usual care controls [1]. While the effect size between real and sham acupuncture are usually small, most studies do agree that acupuncture does reduce chronic pain [2, 3].

Acupuncture sensation, which is a somatosensory sensation induced by acupuncture needling, has been considered an important factor contributing to therapeutic effects in acupuncture treatment [4]. A recent study found that greater pain relief (analgesic effect on thermal pain) was accompanied by greater acupuncture sensation (in numbness and soreness), and concluded that acupuncture sensation is a useful indicator of the clinical efficacy of acupuncture treatment [5]. In another study, greater analgesic effect (e.g., increased pressure pain threshold) was also reported in a treatment group with higher acupuncture needling sensation [6]. While several studies now suggest this link between needling sensation and clinical efficacy [7], other studies have not found associations between acupuncture sensations and analgesia [8], and controversy remains suggesting the need for more thorough research on this topic [9]. Acupuncture sensation may differ from evoked pain sensation, in terms of brain response, as suggested by neuroimaging studies [10–13]. Thus, acupuncture analgesia may differ from classical conditioned pain modulation [14].

Significant inter-subject heterogeneity in acupuncture treatment response has been noted and may be at least partially related to patients’ coping strategy toward acupuncture and/or their clinical pain. For instance, a recent study [15] showed that acupuncture treatment in patients with chronic musculoskeletal pain enhanced their coping towards pain (e.g. positive reframing) as well as reducing pain intensity. Another study [16] emphasized the positive influence of acupuncture’s long-term effect on cognitive and emotional pain coping in chronic low back pain patients. It is also possible that coping strategy can modulate clinical outcomes. For instance, Koh et al. [17] suggested using a treatment approach based on the individual’s coping strategy for the effective treatment of cancer patients. For chronic pain, rheumatoid arthritis patients using a passive coping strategy also reported higher levels of clinical pain intensity [18]. In evoked, experimental pain studies, Hayes et al. used a cold pressor task and demonstrated greater pain tolerance for an acceptance coping approach [19], while Keogh et al. [20] showed gender differences in cold pain ratings under different coping instructions (emotion- and sensory-focused coping). These results emphasize the impact of coping strategy on pain modulation. We propose that pain and somatosensory processing may also be modulated by a patient’s coping strategy toward acupuncture needling, which could produce clinically relevant effects by modulating acupuncture sensation.

Acupuncture sensation includes somatosensory sensations such as numbness and dull pain, evoked by acupuncture needling. We hypothesized that different coping strategies (active versus passive) towards acupuncture needling can modulate clinically-relevant somatosensory and pain thresholds, as well as the linkage between acupuncture sensation and such thresholds. We devised an experimental paradigm to induce active and passive coping to acupuncture needling. Quantitative sensory testing (QST) was performed to evaluate the sensory threshold change for various somatosensory modalities [21], while autonomic response to needling was estimated to explore the influence of coping on acupuncture [22] and physiological response [23], and how these factors relate to important QST variables.

## Methods

The experiment consisted of quantitative sensory testing (QST) sessions before and after electro-acupuncture (EA) stimulation. Subjects were randomly assigned to either an active or passive coping strategy group. QST was performed to investigate pain and sensory threshold changes induced by EA. During EA stimulation, heart rate (HR) and skin conductance (SC) were measured to evaluate physiological response under different psychological (active/passive coping) strategies for coping with EA stimulation.

### Subjects

A total of 61 healthy volunteers (22.3 ± 2.6 years old, μ ± σ) took part in the study. The subjects were recruited via e-mail advertisements, adhering to the guidelines of Kyung Hee University for the distribution at the neighbouring institutions. We excluded subjects reporting pain and autonomic/psychological disorders (e.g., depression or anxiety), and showing difficulties in sensory perception and recognition. All the participants submitted a written informed consent in accordance with the Helsinki Declaration. The protocol of our study was submitted to, and approved by the Institutional Review Board (ethics committee) of Kyung Hee University (KHU IRB 2010–012, Additional file 1). To test for any baseline differences in terms of attitudes toward acupuncture and expectation of acupuncture sensations between the AC and the PC groups, subjects completed adapted versions of questionnaires pertaining to perception of bodily sensations [24], belief in the effectiveness of acupuncture treatment [25] and expected acupuncture sensations [4]. Previous acupuncture experience was also reported by subjects.

### Electroacupuncture (EA) and experimental coping conditions

The subjects were randomized into active coping (AC) or passive coping (PC) strategy groups (i.e. parallel-group study, allocation ratio = 50:50, Figure 1). For both the AC and PC groups, acupuncture needles (sterilized stainless steel, 0.25 × 30 mm, DongBang Acupuncture, South Korea) were inserted at two acupoints (ST36 and GB39, Figure 2C) in the left lower leg by an experienced acupuncturist (KP). The needles were then manipulated briefly to induce acupuncture sensation. EA was then applied to the needles using a constant-current stimulator (STN-100, StraTek, South Korea). Electrical pain threshold was determined prior to the EA stimulation (Figure 2A, Electrical Pain Threshold test at ST36 and GB39), and electrical current intensity for EA stimulation was set slightly below pain threshold (95% of the individual’s electrical pain threshold, i.e., strong but not painful stimulation). EA stimulation was applied at 16 Hz, following prior animal studies demonstrating that EA stimulation at this frequency produces endogenous opioid-mediated analgesia via μ-, δ-, and κ-receptors in the central nervous system [26]. For EA stimulation, eight discrete blocks of EA stimuli were applied (stimulation duration = 6 sec, ISI = 60 sec, total length = 480 sec, Figure 2B) using the previously defined stimulus frequency and current intensity.

**Figure 1:**
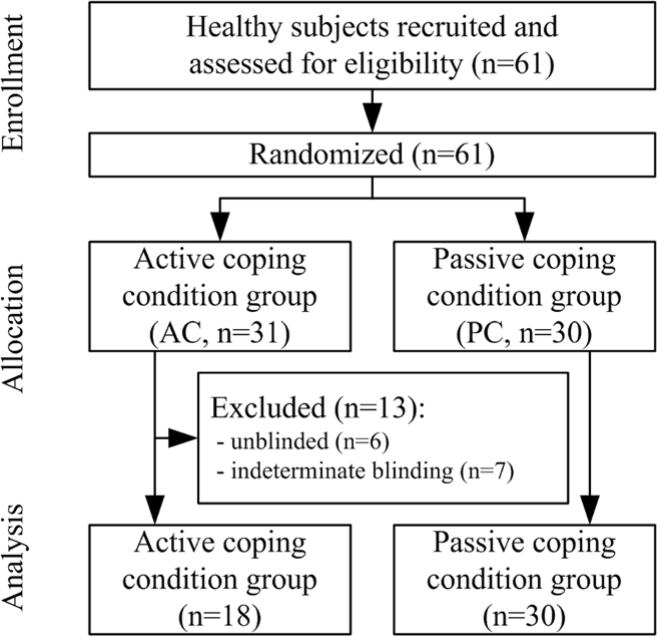
**Flow diagram of the study.** Recruited healthy subjects were randomized into active and passive coping groups. Successfully blinded subjects in the active coping group were compared with subjects from the passive coping condition group.

**Figure 2:**
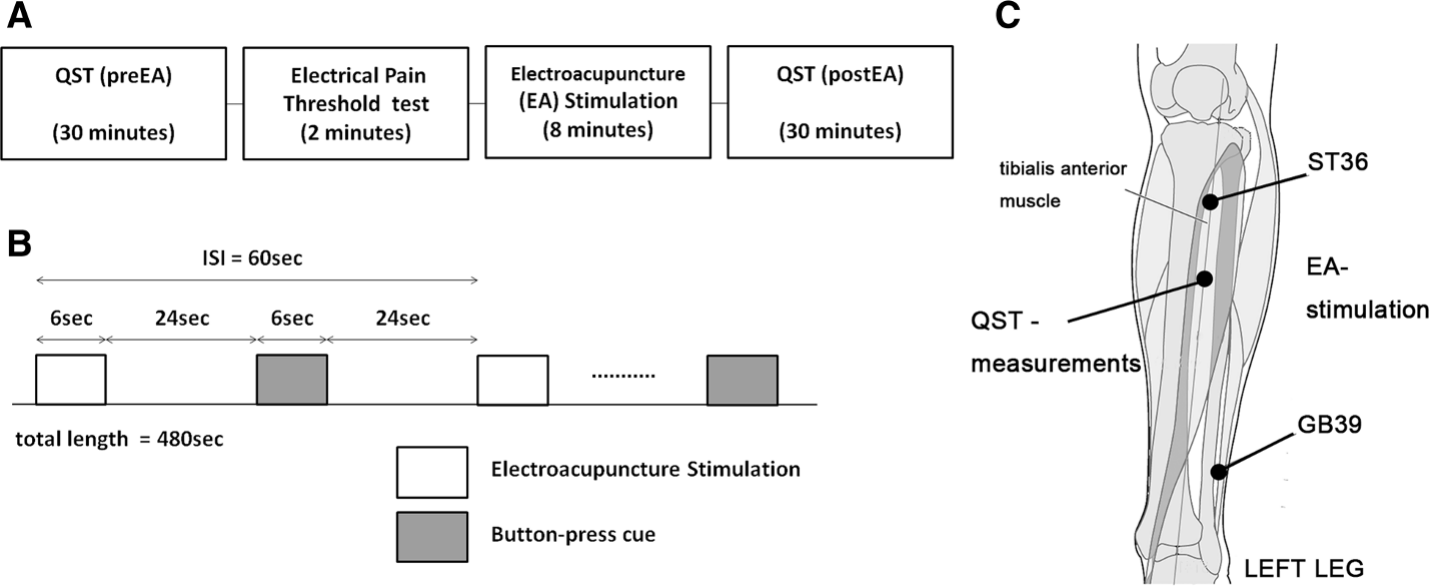
**Experimental design. A**: Overview of the experiment procedure. Two 30 minute QST sessions were performed before and after an 8-minute electroacupuncture stimulation procedure. **B**: Experimental paradigm of the electroacupuncture (EA) stimulation procedure. EA stimulation blocks preceded button-press cues, which were important for inducing active and passive coping strategies. The AC group was told that EA current intensity would be reduced according to the button press response, while the PC group was told to simply press the button after the cue. **C**: Body locations for EA stimulation (acupoints ST36 and GB39), and quantitative sensory testing (QST-measurements) on the left leg. n.b. ISI = inter-stimulus interval.

Subjects were randomly assigned into experimentally-controlled active (AC) or passive (PC) coping strategy groups, which set specific instructions as to how subjects were to cope with the EA stimuli (Figure 1). During EA stimulation, a visual cue was presented 30 seconds after each EA stimulus block onset (Figure 2B), and subjects were asked to respond by pressing a button immediately after this visual cue. For the AC group, the subjects were told that EA current intensity (which was initially set just below pain threshold) could be reduced if they pressed the button twice, and would not if they pressed it once. In reality, however, the EA current intensity was not reduced in either case. Thus, subjects in the AC group thought that they could control stimulation intensity, and would thus consider the EA stimulus as an ‘escapable stressor’ [27]. For the PC group, subjects were asked to endure the EA stimulation, and simply press the button whenever the visual cue was given. Thus, PC subjects would consider the EA stimulation an ‘inescapable stressor’ [27]. In sum, AC and PC groups differed in the psychological mindset for coping with EA stimulation.

### Quantitative sensory testing (QST)

QST was performed before and after EA to evaluate pain and sensory threshold change by acupuncture under different coping strategies (Figure 2A). Eight parameters were measured, in the following order: vibration detection threshold (VDT), pressure pain threshold (PPT), mechanical pain threshold (MPT), mechanical detection threshold (MDT), warm detection threshold (WDT), cold detection threshold (CDT), heat pain threshold (HPT), and cold pain threshold (CPT) [21]. Each QST parameter was assessed by the same individual over a 30-minute testing session. MPT was assessed by an average of 5 trials, MDT by an average of 10 trials, while other thresholds were assessed by an average of 3 trials. Stimulus location for all thresholds except for VDT (lateral condyle of tibia) was on the same site midway on the anterior left lower leg (Figure 2C). Trials were separated by 30 seconds, ensuring that subjects did not feel any lingering sensations from prior trials. Multiple QST modalities were used in attempt to differentiate effects mediated by different neural pathways (e.g. Aβ, Aδ, and/or C-fibers) [21].VDT was assessed using a 64-Hz tuning fork (Rydel Seiffer tuning fork, Germany). The tuning fork was placed upright over a bony prominence at the lateral condyle of the tibia and was left in place until the subject reported loss of sensation. The minimum magnitude of vibration (0–8 scale visible on the fork, 0: strong, 8: none) reported as being felt by the subject was recorded as a threshold for each trial. A pressure algometer (FDX 50, Wagner Instruments, USA) was used to evaluate the PPT at the QST measurement site (Figure 2C). The pressure was applied at approximately constant velocity of 1 kgf/sec, until the subject reported discernible pain onset. For each trial, the minimum magnitude causing pain was recorded as PPT. The MPT was measured similarly for each trial using the same algometry device, but with a fine, blunt tip to elicit a pinprick pain. The MDT was acquired by averaging over ten trials (five with ascending and another five with descending order) with pressures ranging from 0.02 to 60 g delivered via von Frey monofilaments (Touch-Test Sensory Evaluator Kit, North Coast Medical Inc., USA). The thermal thresholds were measured using a thermal stimulation device (PATHWAY, Medoc, Israel). A thermode (3 cm × 3 cm) was placed on the skin surface of the QST measurement site (Figure 2C). The temperature was then increased or decreased from 32°C at a rate of 1°C per second. For detection thresholds, subjects were asked to press a button when they noticed the temperature of the thermode changing form baseline to warmth or cold (WDT, CDT). For pain thresholds, subjects were asked to press a button once heat or cold sensation became painful (HPT, CPT). The temperature was recorded immediately after button press, and was used in the multi-trial average as the subject’s threshold.

Changes in pain and sensory detection thresholds (ΔVDT, ΔPPT, ΔMPT, ΔMDT, ΔWDT, ΔCDT, ΔCPT, and ΔHPT) were calculated as the post – pre EA difference score (paired *t*-test). In order to more easily interpret correlations, the ΔCPT and ΔCDT scores were inverted.

Because of device unavailability, not all thresholds could be acquired from all subjects (data collected for AC group: n = 15 for CDT, WDT, HPT, and CPT measurement, and n = 3 for MDT and MPT measurement; PC group: n = 15 for CDT, WDT, HPT, and CPT measurement, and n = 12 for MDT and MPT measurement; VDT and PPT were collected from all the subjects). Due to the low number of AC subjects contributing MPT and MDT data, these measures were not used for further analyses.

### Autonomic and psychophysical response to EA

To evaluate the influence of active and passive coping strategy on physiological response, peripheral autonomic activity was estimated by HR and SC responses to EA stimuli [27]. Physiological signal was collected using a data acquisition device (PowerLab/800, ADInstruments Inc., Australia) and a 1 kHz sampling rate. The HR responses were calculated from the electrocardiogram, which was collected with three Ag/AgCl electrodes (Kendall, Covidien, USA). Before processing, ECG data were notch filtered at 60 Hz (ML132, BIO Amp, ADInstruments Inc., Australia). The SC signal was collected from the index and middle fingers of the left hand (ML116, GSR Amp, ADInstruments Inc., Australia).

HR and SC data were analyzed based on EA stimulation onset, and EA device operation was synchronized with autonomic data acquisition using a common TTL pulse generator. HR and SC responses were first normalized by subtracting the average value of the 4-second baseline period immediately preceding stimulus onset. Following normalization, the maximum HR decrease in the 0–8 second post-stimulus window and maximum HR increase in the 8–16 second post-stimulus window [28] were calculated. The amplitude of SC response in the 0–8 second post-stimulus window [29] was also calculated (MATLAB 7.10, The MathWorks Inc., Waltham, MA, USA). Acquisition artifacts and equipment error led to loss of physiological data from 6 subjects in the AC group, and 7 in the PC group.

In order to quantify the intensity of acupuncture-induced sensations, the MGH Acupuncture Sensation Scale (MASS) was completed by all subjects following EA stimulation (0–10 scale for all sensations, including aching, soreness, deep pressure, heaviness, fullness, tingling, warmth, numbness, dull and sharp pain etc.) [4]. The MASS Index (MI) was calculated from the weighted sum of MASS items pertaining to deqi sensation [4]. Additionally, in order to assess potential habituation or sensitization in acupuncture sensation over the EA stimulation period, we divided the EA stimulation session into two equal-duration time intervals, an early 4-minute interval and a late 4-minute interval. Following the EA session, subjects reported general acupuncture sensation intensity (0–10 VAS scale) for both the early and late 4-minute periods. The change from the first to the second phase was calculated to test for habituation (negative values) or sensitization (positive values). Acupuncture sensation report was not able to be collected from one subject in the AC group.In addition, an in-depth interview for blinding was performed retrospectively to evaluate whether subjects in the AC group believed they were following an active coping strategy. If subjects in the AC group reported that stimulation intensity was indeed reduced on demand (regardless of exact sensation intensity), these subjects were considered to be adequately blinded, and their data were used in subsequent analysis. If subjects did not press the button twice to reduce EA current intensity, adequate blinding could not be determined and data were considered separately (Figure 1, indeterminate blinding).

### Statistical analysis

Two-tailed Student’s *t*-test (SPSS v. 10.0.7, Chicago, IL, USA) was used for within-group (i.e., paired *t*-test) and between-group (unpaired *t*-test) analysis, and the difference at 95% confidence level (P < 0.05) was considered statistically significant. For a cross-correlation analysis, Pearson’s correlation coefficients were calculated between parameters and considered as significant at P < 0.05. As this was an exploratory pilot experimental study, p-values between 0.05 and 0.10 were reported as trending significance.

## Results

For subjects assigned to the active coping condition (n = 31), 18 subjects were deemed to be adequately blinded and, thus, included in the analysis; 7 subjects pressed the button only once (i.e. indeterminate blinding) in AC group, and among the rest (n = 24) who pressed the button twice, 6 were found to be unblended (Figure 1). The AC (n = 18, 16 males) and PC (n = 30, 23 males) groups did not show any differences in age (AC = 21.8 ± 3.2, μ ± σ, years old, PC = 22.2 ± 2.4 years old), perception of bodily sensations, belief in the effectiveness of acupuncture treatment, or previous experience with acupuncture treatment. Greater expectation of acupuncture sensations was reported by AC compared to PC (MASS Index: AC = 5.73 ± 1.55, PC = 4.49 ± 2.02, μ ± σ, P < 0.05); however, it was not significantly correlated to any QST measure or autonomic response to EA.

### QST response to EA stimulation under active and passive coping conditions

Following EA, subjects in the AC group showed reduced pressure pain threshold (ΔPPT = -0.72 ± 1.33 kPa, μ ± σ, P < 0.05) and cold pain threshold (ΔCPT = -1.87 ± 3.39°C, P = 0.05). In other words, after EA, reduced pressures and higher temperatures were rated as painful. No change was observed in the other QST parameters (VDT, CDT, WDT, HPT, MDT and MPT) for AC. Several cross-modal QST measures showed consistent, correlated change following EA for AC. Specifically, subjects who reported reduced PPT (decreased pressure), also reported worse CPT (increased temperature) (r = 0.57, P < 0.05). Additionally, increases in WDT were correlated with increases in HPT (r = 0.61, P < 0.05).

There was no significant change in any QST parameter following EA in the PC group. However, we did note cross-modal QST change correlations across PC group subjects. Specifically, PPT change was correlated with change in MPT (r = 0.83, P < 0.001), and was negatively correlated with change in WDT (r = -0.63, P < 0.05).

### Autonomic response to EA stimulation

**Figure 3:**
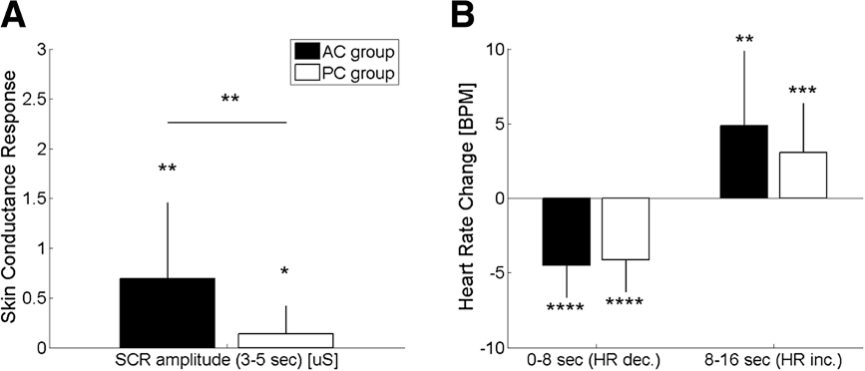
**SC and HR response to EA stimulation in the active and passive coping condition groups. A**: SC response amplitude showed a greater increase in the AC group compared to the PC group, suggesting increased sympathetic activation under AC. **B**: HR response was similarly biphasic (early decrease followed by later increase) was similar for both groups. n.b. *< 0.05, **< 0.01, ***< 0.001, ***< 0.0001. The error bars indicate the standard deviation.

For both AC and PC, the SC response time-course increased 3 seconds after EA stimulation block onset, suggesting an increase of sympathetic activity. However, SC response amplitude (3–5 sec), was greater (p < 0.01) for AC (0.69 ± 0.77 μS, μ ± σ) compared to PC (0.14 ± 0.28 μS, Figure 3A).For both AC and PC, HR decrease was observed within 8 seconds after EA stimulus onset. HR then increased above baseline values 8–16 seconds after stimulus onset (Figure 3B). These changes in HR did not differ between AC and PC groups.

### Psychophysics during EA stimulation

There were no differences between AC and PC in acupuncture sensation reported following EA stimulation (e.g., fullness: AC = 4.6 ± 2.5, PC = 4.8 ± 2.5, μ ± σ, P = 0.80, tingling: AC = 4.4 ± 2.1, PC = 4.0 ± 2.7, P = 0.62, dull pain: AC = 4.9 ± 1.6, PC = 4.4 ± 2.6, P = 0.44, see Table 1). However, in order to assess potential habituation or sensitization in acupuncture sensation over the EA stimulation period, we calculated the difference between acupuncture sensation ratings for a late versus early 4-minute EA stimulation interval. On average, subjects reported a positive intensity change (sensitization) for the AC group, and a negative change (habituation) for the PC group, with trending significance for the inter-group difference (AC: ΔVAS = 0.69 ± 2.84, PC: ΔVAS = -0.84 ± 2.60, μ ± σ, AC vs PC p-value = 0.06).

**Table 1:** **Acupuncture-induced sensations to EA stimuli in the AC and PC groups**

Sensations	AC group	PC group	P-value
Aching	5.73 ± 1.55	4.49 ± 2.02	0.32
Soreness	4.41 ± 2.00	3.17 ± 2.64	0.10
Deep pressure	6.06 ± 1.52	5.40 ± 2.06	0.26
Heaviness	6.06 ± 1.75	5.63 ± 2.34	0.52
Fullness	4.65 ± 2.47	4.83 ± 2.45	0.80
Tingling	4.41 ± 2.09	4.03 ± 2.72	0.62
Warmth	2.70 ± 2.17	1.67 ± 1.67	0.07
Cold	1.94 ± 1.39	1.90 ± 2.05	0.94
Numbness	4.76 ± 2.05	4.10 ± 2.62	0.37
Dull pain	4.94 ± 1.56	4.40 ± 2.59	0.44
Throbbing	3.59 ± 1.84	3.13 ± 2.57	0.52
Sharp pain	3.18 ± 1.33	2.80 ± 2.01	0.49
Spread	4.41 ± 1.94	4.83 ± 2.45	0.55
MASS Index (MI)	6.60 ± 0.91	6.23 ± 1.65	0.39

AC: Active coping group, PC: Passive coping group. Data are shown as μ ± σ.

### Cross-correlation between QST, autonomic activity and acupuncture sensation

**Figure 4:**
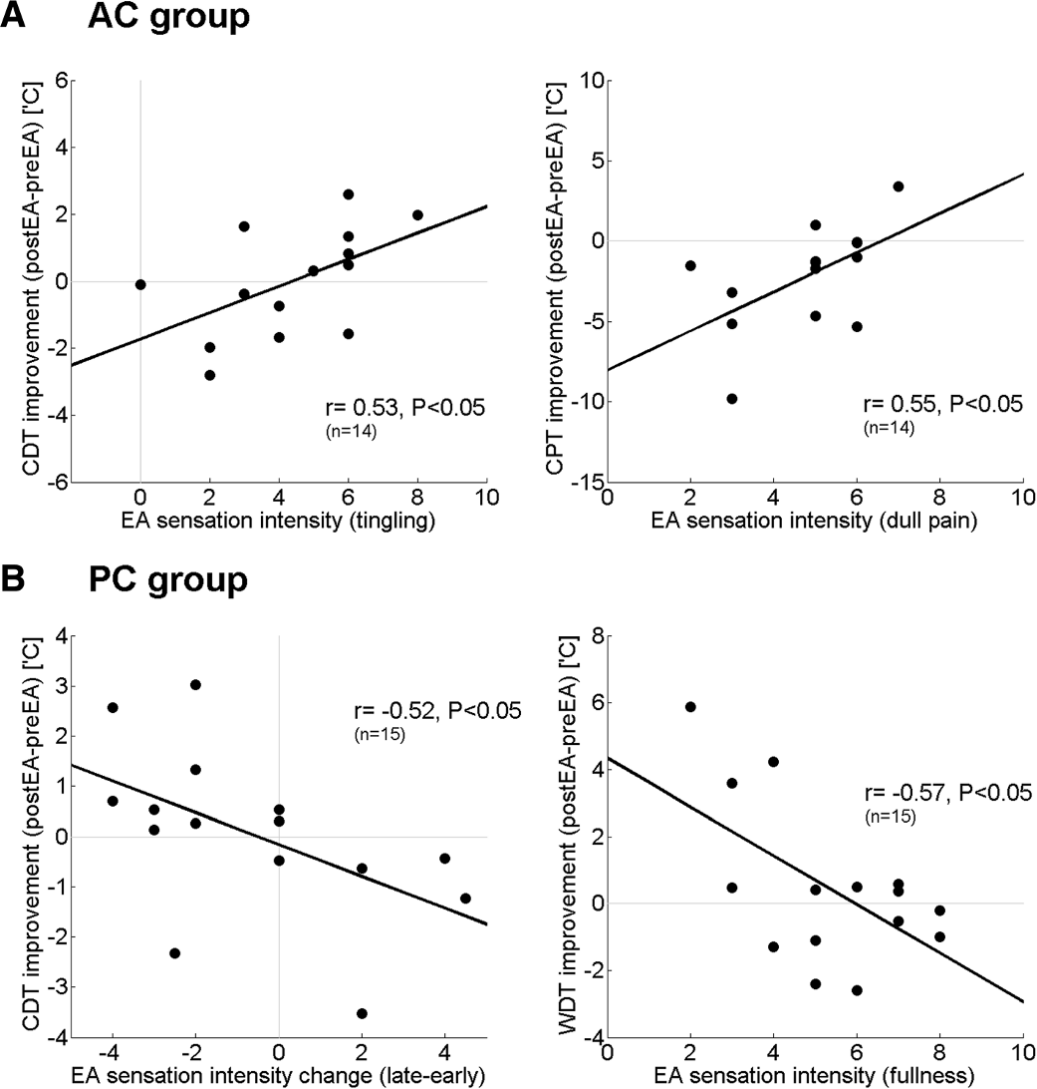
**Cross-correlation between the intensity of acupuncture sensation and the change in sensory detection threshold. A**: For AC, greater acupuncture sensation was associated with improved pain and sensory detection thresholds. **B**: For PC, greater acupuncture sensation and sensation sensitization (greater acupuncture sensation in the late vs. early stimulation period) was associated with reduced sensory detection thresholds.

In the AC group, there was a significant correlation between some QST parameters and specific acupuncture sensations. That is, the improved pain and sensory detection thresholds were associated with stronger acupuncture sensations (ΔVDT vs. MI: r = 0.58, P < 0.05, ΔCDT vs. tingling: r = 0.53, P < 0.05, ΔCPT vs. tingling; r = 0.55, P < 0.05, ΔCPT vs. dull pain; r = 0.55, P < 0.05, Figure 4A). Significant correlation between some QST parameters and autonomic response to EA was also found. Specifically WDT change was positively correlated to SCR amplitude (ΔWDT vs. SCR amplitude in 3–5 sec, r = 0.56, P < 0.05). Thus, worse WDT outcomes (i.e. higher temperatures needed to sense skin warming) were associated with greater sympathetic responses to EA.Interestingly, the opposite results were observed in the PC group; greater improvement of several pain and sensory detection thresholds were associated with weaker acupuncture sensation intensity (ΔVDT vs. heaviness: r = -0.38, P < 0.05, ΔWDT vs. fullness: r = -0.57, P < 0.05, ΔWDT vs. throbbing: r = -0.56, P < 0.05, Figure 4B). Although we should note that change in PPT was positively correlated with sensation intensity (ΔPPT vs. numbness: r = 0.37, P < 0.05). We also noted that greater habituation of sensation (more decreased sensation change between the late and early EA period) was negatively associated with change in CDT (Δintensity vs. ΔCDT: r = -0.52, P < 0.05, Figure 4B). In other words, greater habituation was associated with better detection threshold. Correlation between autonomic and QST measures was also noted. Post-stimulus HR increase (8-16 sec after stimulus onset) was negatively correlated with cold detection threshold (HR increase after 8–16 sec vs. ΔCDT: r = -0.70, P < 0.05).

## Discussion

This study was designed to investigate the influence of cognitive coping strategy during EA stimulation on autonomic response to EA, acupuncture-induced sensations, and QST outcome measures evaluated before and after acupuncture stimulation. EA stimulation was set to relatively strong electrical current intensities, and experimental coping conditions, active and passive coping, were devised to manipulate the subject’s mindset (coping strategy) for how they cope with this stimulation. We found that psychological coping strategy affects autonomic and QST outcomes, and differentially influences the association between acupuncture sensation and improvements or worsening of somatosensory and pain detection thresholds. Specifically, in the AC group, improved pain and sensory detection thresholds were correlated with greater acupuncture sensation, while in the PC group, improved pain and sensory detection thresholds were negatively correlated with greater acupuncture sensation. Our results highlight the influence of coping strategy, a variable rarely controlled in acupuncture research, on autonomic and clinically-relevant QST responses to acupuncture stimulation.

### Experimental manipulation successfully determines coping strategy and produces differential autonomic response

In this study, the active coping condition was devised to make subjects in the AC group consider EA stimulation to be an “escapable stressor”, while the passive coping condition was devised to make subjects in the PC group consider EA to be an “inescapable stressor”. Different verbal instructions were given to change the psychological coping strategy toward the stimulation: the AC group was told that EA stimulation intensity could be decreased after a button press, while the PC group was asked to endure the applied stimulation. In reality, neither group could control EA stimulation intensity.

Using this paradigm, we found that coping strategy could be successfully induced, as many (58%) subjects in the AC group believed that stimulation intensity was being reduced after the button press. Similar experimental paradigms have been applied in humans for aversive auditory stimuli [27], and showed that AC is associated with greater sympathetic activation. Studies in animal models have also reported an association between active coping strategy and sympathetic activation [30]. In our study, we also noted greater sympathetic activation (SC response) to EA in the AC group. This was in spite of the fact that stimulus intensity (in terms of EA current intensity or EA-induced sensation intensity) was not different between AC and PC. Thus, greater sympathetic activation to EA under the AC manipulation likely reflects greater arousal in response to a more salient stimulus, as subjects believe that the intensity of this stimulus can be controlled.

In addition to the human physiological studies on coping strategy by Beh et al. [27] mentioned above, animal studies have investigated brain mechanisms supporting active and passive coping response strategies. Lumb et al. [23] found that active coping for an escapable pain stimulus produces sympathetic activation (pressor response) via L/DL-PAG (lateral/dorsolateral periaqueductal grey) in the brainstem. This was in contrast to passive coping in response to an inescapable pain stimulus, which produces sympathetic deactivation (depressor response) mediated by ventrolateral (VL)-PAG. Thus, AC and PC strategies coordinate different sub-regions of the PAG, suggesting differential neural circuitry via a brain region well-known for its role in pain modulation (i.e. acupuncture analgesia, [31]), and central autonomic control (sympathetic activation to escapable pain via L/DL-PAG, and parasympathetic activation to inescapable pain via VL-PAG) [32].

### Coping strategy modulates QST outcomes

**Figure 5:**
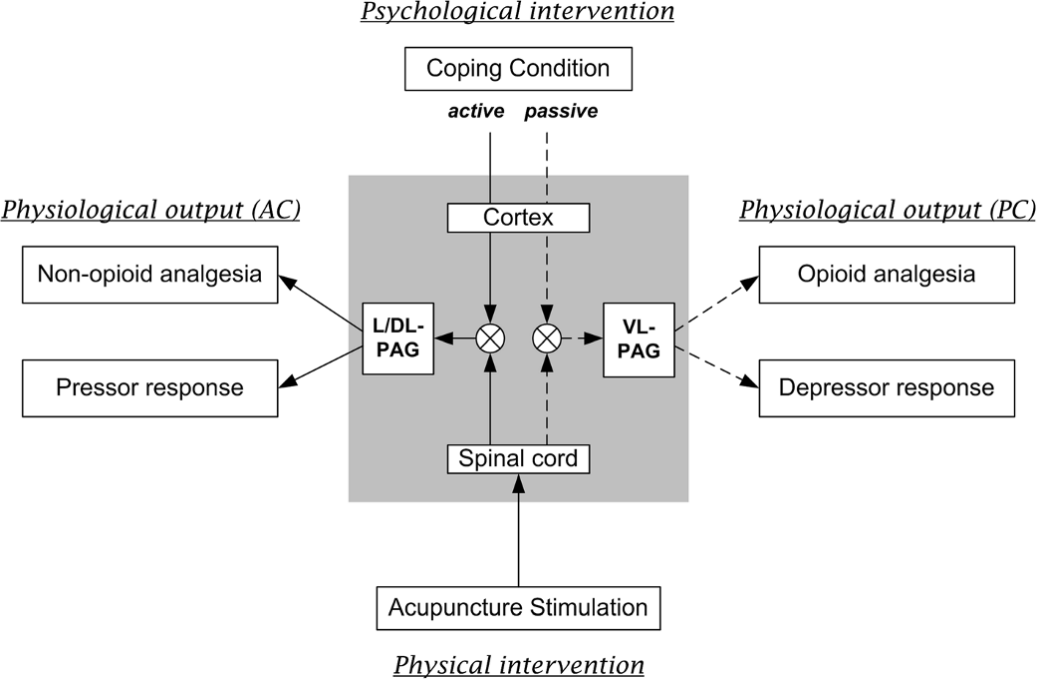
**Neurobiological integrative model.** Our results can be integrated with existing animal models for PAG response to escapable and in-escapable stressors [32]. The psychological coping condition is integrated with physical stimulation intensity, modulating physiological outcomes via different PAG subregions. Specifically, L/DL-PAG activity mediates active coping strategies to impart sympathetic mediated non-opioidergic analgesia, while VL-PAG activity mediates passive coping strategies to impart parasympathetic mediated, opioidergic analgesia.

In order to evaluate pain and sensory threshold response to EA under different coping conditions, QST was performed before and after EA stimulation. Several studies have reported an increase in PPT [14, 21, 33] following EA or manual acupuncture in healthy subjects (without controlling for coping). Interestingly, we found decreased PPT and CPT in the AC (but not PC) group after EA stimulation in this study. This may have been due to differences in experimental protocol between ours and previous studies - e.g. our block-design 8-minute EA stimulation procedure may have been too short for inducing QST improvements under PC. For AC, prolongation of an increased sympathetic response to EA (greater SCR for AC) may have led to augmented descending facilitation and decreased pain thresholds via cortical and midbrain influences. Additionally, reduced pain thresholds and hyperalgesia have been reported for low doses of opioid usage (i.e., opioid-induced hyperalgesia [34]) in both healthy subjects and chronic pain patients, and may be mediated by glutamate and NMDA activity [35]. Such hyperalgesic effects might also be produced by specific PAG activity. L/DL-PAG, which supports active coping and non-opioid analgesia, is known to inhibit VL-PAG which supports passive coping and opioid analgesia [36]. Thus suppressed opioid analgesic effect of VL-PAG by increased L/DL-PAG activity might support both low-dose opioid-induced hyperalgesia and AC-induced PPT and CPT decrease (see Figure 5 for summary).

### Coping strategy differentially affects the association between acupuncture sensation and QST outcomes

Interestingly, acupuncture sensation was differentially related to pain and sensory detection thresholds, depending on coping strategy. In the AC group, higher acupuncture sensation intensity elicited improved pain and sensory detection thresholds (VDT, CDT, and CPT). However, in the PC group, higher acupuncture sensation intensity was associated with reduced sensory detection thresholds (VDT, CDT and WDT). In addition, increased and decreased shifts in sympathovagal balance (observed in HR response) were correlated with pain and sensory modulation in the AC and PC groups, respectively. These results further support PAG involvement, as this brain region relates to pain modulation, emotional coping, and autonomic responsivity. In fact PAG plays a crucial role in pain modulation following external pain-like stimuli via descending inhibitory control (arcuate nucleus of the hypothalamus – PAG – nucleus raphe magnus – spinal cord), such as that following acupuncture analgesia [31]. Interestingly, different subregions of the PAG produce analgesic effects via different mechanisms. That is, L/DL-PAG produces non-opioid mediated analgesia, while VL-PAG produces opioid mediated analgesia [32]. In our study, active coping coupled with high acupuncture sensation intensity may synergistically lead to greater L/DL-PAG activation, leading to greater sympathetic outflow. Inversely, passive coping coupled with low stimulation intensity may serve to activate VL-PAG, resulting in reduced sympathetic dominance and an opioid-mediated analgesic effect. Thus, according to these observations, synergistic effects between the psychological coping strategy and the physical intensity of acupuncture stimulation on PAG-mediated control of analgesic and autonomic response can be suggested. Future neuroimaging studies should evaluate such neural-based hypotheses more directly.

Another recent study [37] measured pain tolerance with different experimental coping conditions of acceptance and distraction, and different threat levels (high and low orienting information) for the cold pressor test. The authors reported that the acceptance group with lower threat information against cold stimulation showed higher pain tolerance than the other conditions. The authors also suggest a synergistic effect of psychological and physical sensory conditions on pain modulation, where acceptance coping may be considered similar to a passive coping strategy group, while expectations of low pain sensation may be related to expectations produced by low acupuncture sensation intensity in this study. Taken together, these results support the findings in our study – i.e. improved sensory and pain modulation effects can be achieved for acupuncture stimulation by applying high stimulation intensity in the active coping strategy group and low stimulation intensity in the passive coping strategy group.

From a clinical view point, based on our findings, acupuncture analgesia may be enhanced or weakened according to patients’ coping strategy during treatment. Thus, the instructions given to the patient before and during the treatment (as a psychological intervention) in addition to applied acupuncture intensity (as a physical intervention), are important for symptom improvement and can be thought of as part of the doctor-patient relationship.

Several limitations to our study should be mentioned. First, while we indirectly speculate as to the involvement of the PAG in our effects, PAG activity was not measured directly. Second, based on the results, it was assumed that the cognitive (coping strategy according to the experimental coping condition) and physical (stimulation intensity) interventions have a synergistic effect on the pain processing system, but it is not clear at which level the synergistic effect occurred. The experimental coping manipulation can be implemented in a neuroimaging context, and thus both of these limitations can be addressed in a follow-up neuroimaging study. Third, as this pilot study did not assess state or trait anxiety in our healthy subjects, we were not able to assess the influence of anxiety on physiological or QST outcome measures. Future studies should also specifically evaluate the influence of anxiety on these outcomes. Lastly, our pilot study may have been underpowered for significant effects in some parameters. Future studies should consider the significant inter-subject variability when determining sample size and which specific parameter should serve as primary outcome.

## Conclusions

The novel approach presented in this study suggests that the interaction between psychological coping strategy and acupuncture sensation intensity can differentially modulate pain and sensory detection threshold response to EA. In a clinical context, our findings suggest that instructions given to the patient before and during the treatment can significantly affect therapeutic outcomes and the relationship between acupuncture intensity and clinical response. Specifically, high stimulation intensity coupled with an active coping strategy and low stimulation intensity coupled with a passive coping strategy are recommended for improved therapeutic effects.

## Electronic supplementary material

Additional file 1: **CONSORT 2010 checklist.** (DOC 216 KB)

## Abbreviations

EA: Electroacupuncture
AC: Active coping
PC: Passive coping
QST: Quantitative sensory testing
VDT: Vibration detection threshold
MDT: Mechanical detection threshold
WDT: Warm detection threshold
CDT: Cold detection threshold
PPT: Pressure pain threshold
MPT: Mechanical pain threshold
HPT: Heat pain threshold
CPT: Cold pain threshold
HR: Heart rate
SC: Skin conductance
SCR: Skin conductance response
MI: MGH acupuncture sensation scale index
PAG: Periaqueductal grey.
